# Pulmonary Vasodilator Therapy in Children with Single Ventricle Physiology: Effects on Saturation and Pulmonary Arterial Pressure

**DOI:** 10.1007/s00246-020-02424-w

**Published:** 2020-07-30

**Authors:** Ida Jeremiasen, Karin Tran-Lundmark, Nikmah Idris, Phan-Kiet Tran, Shahin Moledina

**Affiliations:** 1 Department of Experimental Medical Science, Lund University, BMC C12, 221 84 Lund, Sweden; 2 The Pediatric Heart Center, Skane University Hospital, Lund, Sweden; 3 Great Ormond Street Hospital for Children, London, UK; 4 University College London, London, UK

**Keywords:** Single ventricle physiology, Children, Vasodilators, Pulmonary vascular resistance

## Abstract

In children with single ventricle physiology, increased pulmonary vascular resistance may impede surgical progression or result in failing single ventricle physiology. The use of pulmonary vasodilators has been suggested as a potential therapy. However, knowledge on indication, dosage, and effect is limited. A retrospective case notes review of all (*n* = 36) children with single ventricle physiology, treated with pulmonary vasodilators by the UK Pulmonary Hypertension Service for Children 2004–2017. Therapy was initiated in Stage 1 (*n* = 12), Glenn (*n* = 8), or TCPC (*n* = 16). Treatment indications were high mean pulmonary arterial pressure, cyanosis, reduced exercise tolerance, protein-losing enteropathy, ascites, or plastic bronchitis. Average dose of sildenafil was 2.0 mg/kg/day and bosentan was 3.3 mg/kg/day. 56% had combination therapy. Therapy was associated with a reduction of the mean pulmonary arterial pressure from 19 to 14 mmHg (*n* = 17, *p* < 0.01). Initial therapy with one or two vasodilators was associated with an increase in the mean saturation from 80 to 85%, (*n* = 16, *p* < 0.01). Adding a second vasodilator did not give significant additional effect. 5 of 12 patients progressed from Stage 1 to Glenn, Kawashima, or TCPC, and 2 of 8 from Glenn to TCPC during a mean follow-up time of 4.7 years (0–12.8). Bosentan was discontinued in 57% and sildenafil in 14% of treated patients and saturations remained stable. Pulmonary vasodilator therapy was well tolerated and associated with improvements in saturation and mean pulmonary arterial pressure in children with single ventricle physiology. It appears safe to discontinue when no clear benefit is observed.

## Introduction

Total cavopulmonary connection (TCPC) is the final palliative surgical procedure for children with single ventricle physiology. Adequate pulmonary blood flow in these patients is dependent on a low pulmonary vascular resistance. High resistance may hinder surgical progression to total cavopulmonary connection and is known to increase short-term mortality after this surgery [1–3].

Clinical signs and secondary effects of elevated pulmonary vascular resistance include cyanosis, reduced exercise tolerance, protein-losing enteropathy, and plastic bronchitis. Mechanisms such as passive non-pulsatile flow and cyanosis/hypoxemia caused by systemic-to-pulmonary venous collaterals have been suggested to contribute to vascular remodeling and increased resistance over time in a patient with single ventricle physiology [4–6].

In children with pulmonary arterial hypertension, pulmonary vasodilator therapy has been shown to reduce pulmonary vascular resistance and alleviate symptoms [7–9]. In clinical guidelines, children with single ventricle physiology are identified as a group who can suffer from clinically significant pulmonary hypertensive vascular disease even when their mean pulmonary arterial pressure is below 20 mmHg, the threshold of pulmonary hypertension [10–13]. The recently revised pediatric pulmonary hypertension guidelines added patients with single ventricle physiology to group 5 (5.4), pulmonary hypertension with unclear and/or multifactorial mechanism. It also acknowledges that there is insufficient data to show that targeted therapies are safe and efficient in this population and that further studies are required [11].

The two most commonly used oral medications for pulmonary vasodilatation in children are sildenafil and bosentan. Dysfunction of the endothelial nitric oxide pathway has been demonstrated in single ventricle physiology patients, and the lack of pulsatile blood flow has been hypothesized to cause an increase in endothelin-1 [14, 15]. This has provided the rationale for attempts to target these pathways to reduce pulmonary vascular resistance in patients with single ventricle physiology.

Improved exercise tolerance following sildenafil treatment has been reported in patients with total cavopulmonary circulation [16]. Studies have also shown that pulmonary vasodilators lowers mean pulmonary arterial pressure and vascular resistance [17–20]. Sildenafil treatment for up to two years has been reported in patients with single ventricle physiology with no major side effects [21]. Still, data are limited to few studies with small cohorts and/or short-term follow-up, and it is not yet clearly determined if pulmonary vasodilators can provide sustained benefit to patients in TCPC or patients in earlier stages of this surgical pathway.

The aim of this analysis was to investigate the effects of oral pulmonary vasodilators, used as single or combination therapy, in children at different surgical stages of single ventricle physiology. Focus was on potential impact on saturation, pulmonary arterial pressure, and progression to next surgical stage. We also report the incidence of serious adverse events during treatment and at weaning.

## Material and Methods

### Patients

The medical records of children referred to the United Kingdom Service for Pulmonary Hypertension in Children at Great Ormond Street Hospital in London were reviewed. All children < 18 years of age with single ventricle physiology who had been treated with pulmonary vasodilator therapy between 2004 and 2017 were selected for retrospective analysis. The patients were divided into three groups depending on surgical stage at treatment initiation: (1) Stage 1 (shunt, Norwood procedure, or banding), (2) Glenn (bidirectional cavopulmonary anastomosis), and (3) TCPC (total cavopulmonary connection).

### Data Collection

All available medical and surgical notes were reviewed. Collected data included dosage of pulmonary hypertension specific therapies, adverse reactions to medication, progress to next surgical stage, comorbidities and other medical therapies. In addition, ejection fractions and atrioventricular valvular functions from cardiac ultrasound examinations, saturations from non-invasive measurements, and mean pulmonary arterial pressures from cardiac catheterizations were collected.

Saturation and mean pulmonary arterial pressure data were collected from 12 months before and up until the initiation of vasodilator treatment. Follow-up saturations were collected from the first 12 month period after treatment initiation, and mean values were calculated for analysis of potential changes from the initial value. Saturations were also collected from the 12 months period that followed discontinuation of vasodilator therapy, as well as the reason for discontinuation. Follow-up mean pulmonary arterial pressures were collected with a mean time interval between treatment initiation and follow-up of 1.3 years. Surgical stage progress, fenestration of the total cavopulmonary connection tunnel, pulmonary arterial interventions, severe infections, or major changes of other medications may confound findings. Follow-up data for saturations and mean pulmonary arterial pressures contaminated with these events were therefore omitted from analysis. Analyzed and reported data in this study are based on cardiac ultrasounds from 35, saturations from 16, and mean pulmonary arterial pressures from 17 of the 36 patients. Data from the case notes conforming to inclusion criteria, and where no confounding factors omitted the patient from analysis, were included in the study.

### Statistics

Continuous variables are shown as mean and range. Saturation and mean pulmonary arterial pressure data were analyzed for normality and normal distribution could be confirmed. Paired Student´s *t-*test was therefore used for analysis of changes in saturation and mean pulmonary arterial pressure. A *p*-value ≤ 0.05 was considered statistically significant. SPSS and Excel were used for statistical analysis.

## Results

### Patient Demographics, Treatment, and Follow-up Time

A total of 36 patients met the inclusion criteria. 14 patients had a dominant right ventricle, 12 patients had a dominant left ventricle, 9 patients had two contributing ventricles, and one was ambiguous (Table 1). Some of the defects were highly complex: 4 patients had interrupted inferior vena cava, 8 patients had atrial isomerism, and 4 patients had dextrocardia. A detailed description of the cardiovascular malformations in the cohort is listed in Table 1.

**Table 1:** Cardiac diagnosis and stage of palliation when started on vasodilator therapy

Functional dominant ventricle	Primary diagnosis	Total no of patients	Interrupted IVC	Atrial isomerism	Dextrocardia	Treatment following STAGE 1	Treatment following GLENN	Treatment following TCPC
Right ventricle	Hypoplastic left heart syndrome (HLHS)	7					1	6
	Double outlet right ventricle (DORV)	5		1	1	2	2	1
	Aortic stenosis	1					1	
	TGA with pulmonary stenosis	1						1
Left ventricle	Double inlet left ventricle (DILV)	6		1		2	1	3
	Tricuspid atresia	4	1			2	1	1
	Pulmonary atresia IVS	2	1	1	1	2		
Mixed ventricle	Unbalanced AVSD	8	2	5	1	3	1	4
	Mitral stenosis, VSD	1					1	
Unknown	Double inlet ambiguous ventricle	1			1	1		
Total		*n* = 36				*n* = 8	*n* = 12	*n* = 16

*IVC* inferior vena cava, *TGA* transposition of the great arteries, *IVS* intact ventricular septum, *AVSD* atrioventricular septal defect, *VSD* ventricular septal defect, *TCPC* total cavopulmonary connection

The cohort consisted of 18 males and 18 females (Table 2). Mean age in Stage 1 was high due to 5 older patients who never progressed in surgical stage due to complex single ventricle physiology. Mean age at initiation of sildenafil and bosentan was 6.6 (0.6–17.6) and 7.9 (0.9–16.0) years, respectively. Mean total follow-up time on sildenafil and bosentan was 5.3 (0.5–12.1) and 4.0 (0.1–12.8) years, respectively. Total follow-up time with the Pulmonary Hypertension Service was 4.7 (0–12.8) years. The patient with a follow-up time of 0 had one visit only, but had been on sildenafil prior to the visit. One patient died and two underwent heart transplantation. None of the patients had significantly reduced ventricular function or atrioventricular valve insufficiency. In the majority of patients (27/35), ventricular function was normal before the initiation of vasodilator therapy and continued to be normal at last follow-up (29/35). 6/35 patients developed a mild atrioventricular valve regurgitation during the study period. None of the regurgitations were considered hemodynamically significant.

**Table 2:** Demographics and details on vasodilator treatment

	STAGE 1 *n* = 12	GLENN *n* = 8	TCPC *n* = 16	All *n* = 36
Age at first visit [years]	5.1 (1.2–12.3)	6.2 (1.1–10.3)	10.6 (3.5–17.2)	7.8 (1.1–17.2)
Gender ratio M/F	M 4/F 8	M 6/F 2	M 8/F 8	M 18/F 18
Follow-up time [years]	6.1 (2.4–12.8)	5.1 (1.8–8.5)	3.4 (0–11.8)	4.7 (0–12.8)
Age at start of sildenafil [years]	5.2 (0.6–14.9) *n* = 12	4.4 (0.9–8.5) *n* = 8	9.2 (3–17.6) *n* = 15	6.6 (0.6–17.6) *n* = 35
Total sildenafil follow-up time [years]	5.7 (2.3–12.1)	6.2 (0.8–10.3)	4.4 (0.5–9.9)	5.3 (0.5–12.1)
Age at start of bosentan [years]	6 (0.9–12.2) *n* = 10	7.3 (3.2–10.9) *n* = 4	11.1 (5.2–16) *n* = 7	7.9 (0.9–16) *n* = 21
Total bosentan follow-up time [years]	5.3 (0.7–12.9)	4.7 (1.5–8.1)	1.2 (0.1–3.2)	4.0 (0.1–12.9)

*TCPC* total cavopulmonary connection

### Risk Factors and Comorbidities

11 of those 16 who required pulmonary artery banding in their Stage 1 did not undergo banding until after 1 month of age (Table 3). 10 patients required enlargement of a restrictive atrial communication, and 2 patients required a total cavopulmonary connection tunnel intervention. 8 patients required a pulmonary artery branch intervention to relieve stenosis, whereof 6 underwent balloon dilatation and stent implantation and 2 surgical arterioplasty. Arrhythmias were found in 10 patients and other comorbidities in 11 patients, as listed in Table 3.

**Table 3:** Risk factors and comorbidities

	STAGE 1 *n* = 12	GLENN *n* = 8	TCPC *n* = 16	All *n* = 36
PA banding total/(> 1 month)	7/(5)	3/(2)	6/(4)	16/(11) (44%/31%)
PA intervention due to stenosis	1	3	4	8 (22%)
ASD intervention	5	3	2	10 (28%)
TCPC tunnel intervention	-	-	2	2 (6%)
Arrhythmia	1 atrial flutter 1 complete AV block	2 SVT 1 junctional rhythm	1 junctional rhythm 1 JET post-op 2 SVT 1 atrial flutter	10 (28%)
Other comorbidities	1 prim ciliary dyskinesia 1 Chr 4 deletion 1 T21/GERD 1 chronic lung disease	1 factor V Leiden 1 CHARGE/laryngomalacia 1 unclear genetic	1 previous DVT 1 GERD 1 stroke/seizures 1 laryngomalacia/asthma	11 (31%)

*PA* pulmonary artery, *ASD* atrial septal defect, *SVT* supraventricular tachycardia, *JET* junctional ectopic tachycardia, *DVT* deep vein thrombosis, *GERD* gastroesophageal reflux disease, *T21* trisomi21, *TCPC* total cavopulmonary connection

### Vasodilator Treatment

**Fig. 1:**
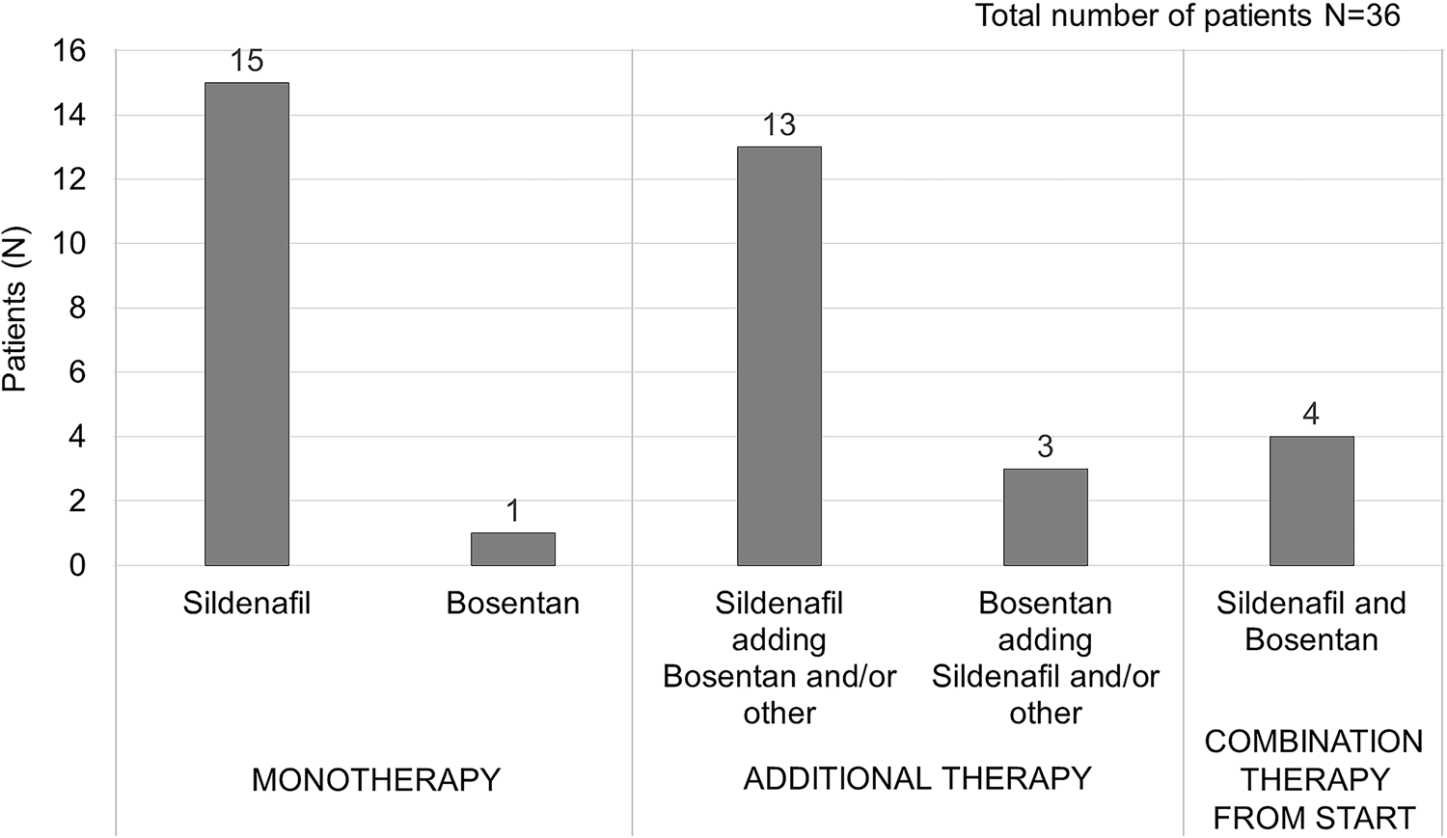
Pulmonary vasodilator therapy. Combination therapy means a combination of two or more vasodilators at some stage during follow-up, usually sildenafil and bosentan

At the time of referral to our center (26/35) 74% of patients treated with sildenafil were already receiving sildenafil and (5/21) 24% of patients treated with bosentan had already commenced bosentan. 16 patients were treated with monotherapy during the study period, whereof 15 with sildenafil and only one with bosentan (Fig. 1). In total 20 patients were treated with combination therapy during the study period. Four of them were on sildenafil and bosentan already at the start of the study, and 16 patients, whose initial therapy was sildenafil (13/16) or bosentan (3/16), had another vasodilator added. The added therapy was bosentan (*n* = 11), sildenafil (*n* = 2), iloprost (*n* = 2), or ambrisentan (*n* = 1). Mean maintenance dose of sildenafil was 2.0 (0.8–7.9) mg/kg/day and of bosentan was 3.3 (1.2–6.1) mg/kg/day. Indication for initiating therapy was elevated pulmonary arterial pressures or in the TCPC group protein-losing enteropathy, plastic bronchitis, cyanosis, or reduced exercise capacity.

### Other Concomitant Therapy

At first assessment, 23 (64%) patients were on diuretics, which also was the most frequently used medication in the cohort besides pulmonary vasodilators (Table 4). 11 (31%) were on aspirin, 17 (47%) on warfarin, 16 (44%) on angiotensin converting enzyme inhibitors, and 5 (14%) on antiarrhythmic medication. At the end of the study no significant changes in concomitant medications were identified for any group. Warfarin was more common in patients with total cavopulmonary connection compared to those in Stage 1 and Glenn.

**Table 4:** Other concomitant therapy

	STAGE 1	GLENN	TCPC	All
Start of study	*n* = 12	*n* = 8	*n* = 16	*n* = 36
Diuretics	6	5	12	23 (64%)
Aspirin	5	5	1	11 (31%)
Warfarin	0	2	15	17 (47%)
ACE inhibitor	2	3	11	16 (44%)
Antiarrhythmics	1	2	2	5 (14%)
End of study	*n* = 7	*n* = 8	*n* = 21	*n* = 36
Diuretics	4	4	11	19 (53%)
Aspirin	7	4	2	13 (36%)
Warfarin	1	3	14	18 (50%)
ACE inhibitor	3	4	11	18 (50%)
Antiarrhythmics	0	2	2	4 (11%)

*ACE inhibitor* angiotensin converting enzyme inhibitor, *TCPC* total cavopulmonary connection

### Change in Saturation

**Fig. 2:**
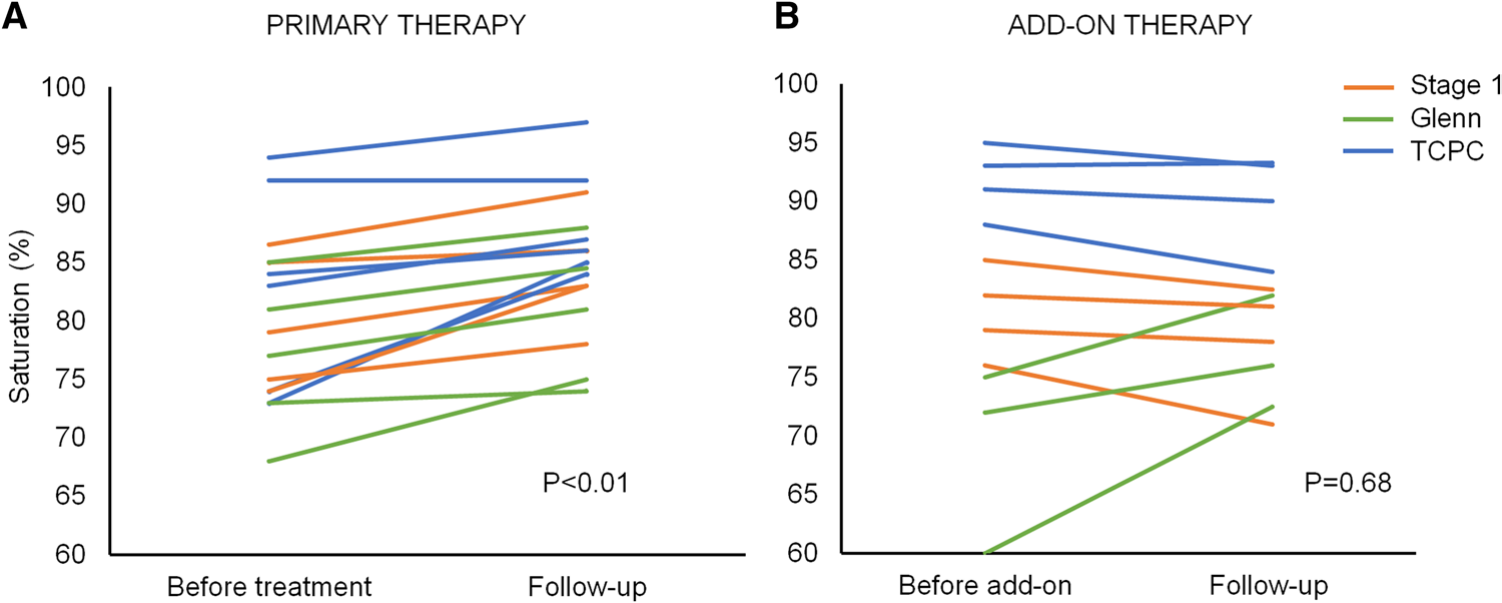
Saturation at treatment initiation and mean values during the first year on therapy. A significant effect of primary therapy is shown in **a**. No significant additional benefit was seen with add-on therapy as shown in **b**. The line indicates stage group at initiation. *TCPC* total cavopulmonary connection

Some patients were already on vasodilator therapy at the time of referral to our center and pre-treatment saturation values were not always included in the referral letters. Complete sets of paired data on saturations, from time of treatment initiation and follow-up with no confounding factors, were available in 16/36 patients. 11 patients had sildenafil, 3 patients had bosentan, and 2 patients had combination therapy. 5 patients were in Stage 1, 5 patients were in the Glenn group and 6 patients were in the total cavopulmonary connection group. Within 12 months after treatment initiation there was a significant improvement in saturation from 80% (SD = 7) to 85% (SD = 6) (*p* < 0.01) (Fig. 2). Later addition of a second vasodilator, bosentan to sildenafil in 11 patients, did not result in any significant additional improvement in saturation (*n* = 11, *p* = 0.68).

### Change in Mean Pulmonary Arterial Pressure

**Fig. 3:**
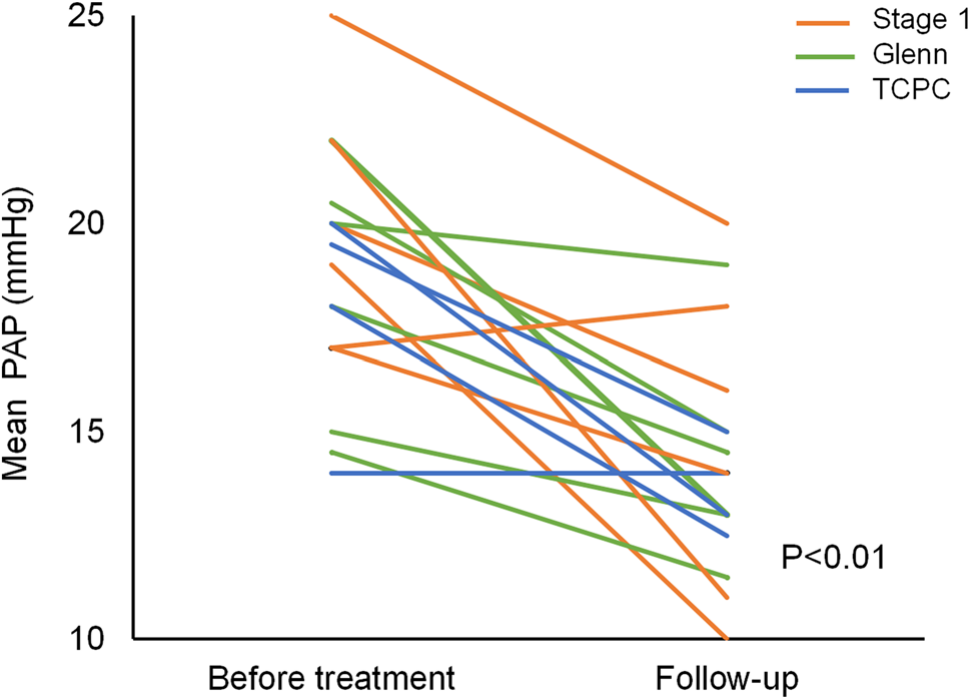
Change in mean PAP (pulmonary arterial pressure) between treatment initiation and at follow-up. The line indicates stage group at initiation. *TCPC* total cavopulmonary connection

All patients had undergone cardiac catheterization, but some pressure measurements were not recorded close enough to the treatment initiation or could not be used because of confounding factors. Complete sets of paired data on mean pulmonary arterial pressure from cardiac catheterization before and mean 1.3 years after treatment initiation were available for 17/36 patients (Fig. 3). 11 patients had sildenafil single therapy, 1 patient had bosentan single therapy, 3 patients had dual therapy from the start, and 2 patients had sildenafil with subsequent add-on bosentan. 7 patients were in stage 1, 6 patients were in the Glenn group, and 4 patients were in the total cavopulmonary connection group. There was significant drop in mean pulmonary arterial pressure from 19 mmHg (SD = 3) to 14 mmHg (SD = 2) (*p* < 0.01). The numbers were too small for subgroup analysis.

### Progress to Next Surgical Stage

**Fig. 4:**
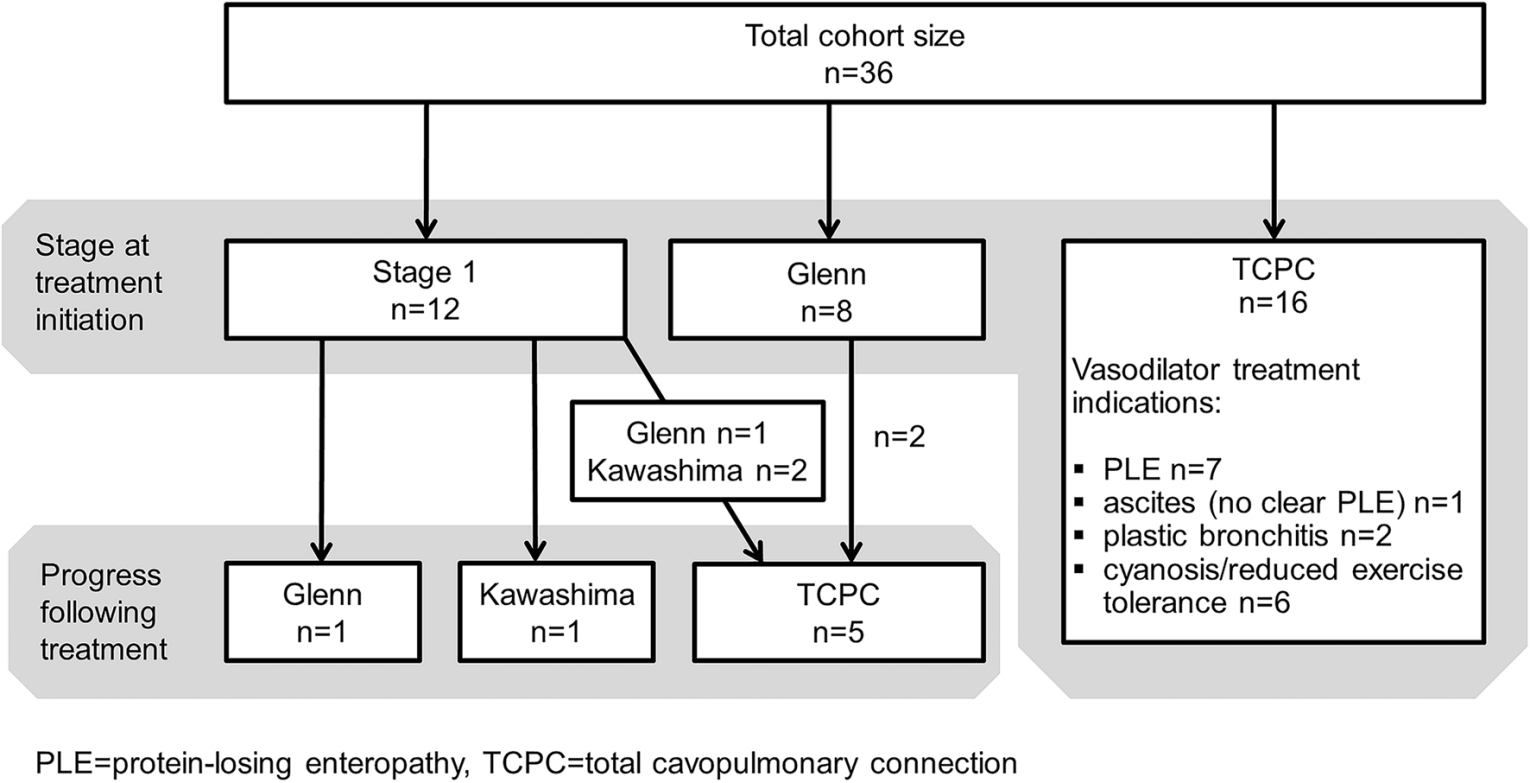
Surgical stage at treatment initiation and surgical progress at follow-up. *TCPC* total cavopulmonary connection, *PLE* protein-losing enteropathy

Before vasodilator therapy initiation 12 patients were in Stage 1 palliation, 8 patients had a bidirectional Glenn shunt, and 16 patients had already undergone total cavopulmonary connection (Fig. 4). At the end of total follow-up time 7 patients had progressed in surgical stage as follows: 1 patient from Stage 1 to Glenn, 1 patient from Stage 1 to Kawashima, 3 patients from Stage 1 to total cavopulmonary connection (whereof 1 patient via Glenn and 2 via Kawashima surgery), and 2 patients from Glenn to total cavopulmonary connection. In total, 17/21 patients with total cavopulmonary connection had a fenestration of the tunnel either at the time of surgery (*n* = 14) or required one at a later stage (*n* = 3).

### Discontinuation of Vasodilator Medication

**Fig. 5:**
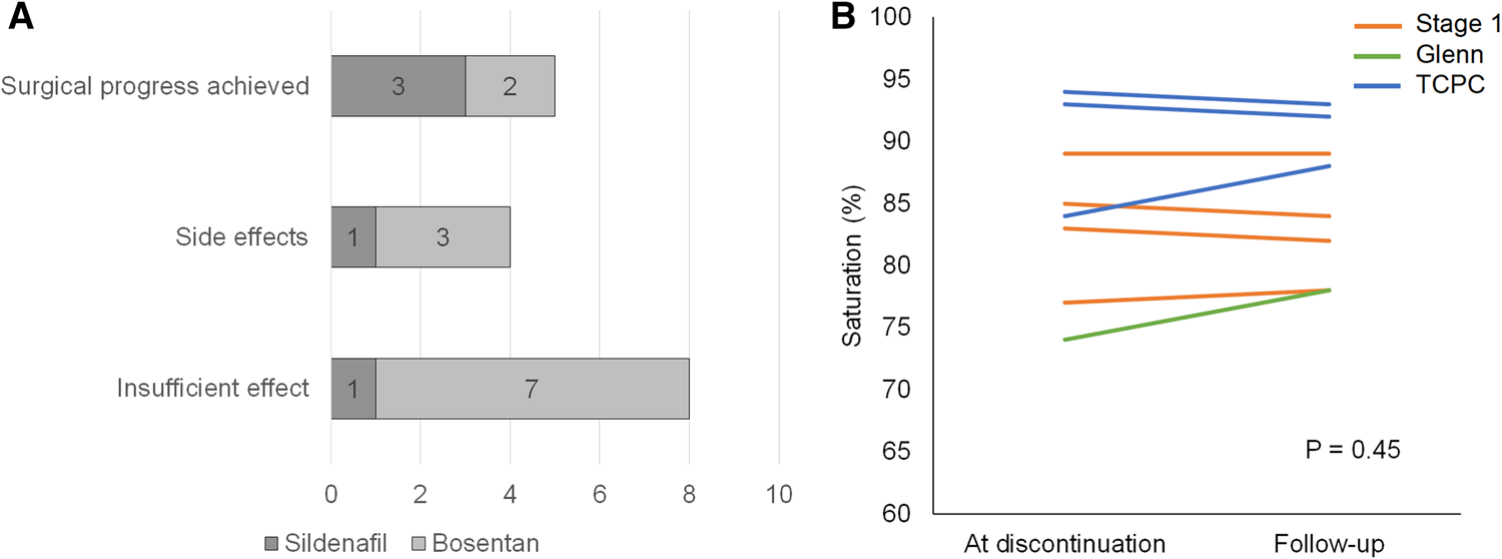
Discontinuation of sildenafil or bosentan. Reason for discontinuation is shown in **a**. No significant change in saturation was seen at follow-up after discontinuation (**b**). The line indicates stage group at initiation

35/36 patients were treated with sildenafil. Discontinuation following progress to next surgical stage with no further need of vasodilators was achieved in 3/35 of sildenafil-treated patients. Another two of sildenafil-treated patients were discontinued, one from the Stage 1 group because of insufficient effect, and one patient from the total cavopulmonary connection group because of priapism. 21/36 were treated with Bosentan. In 7/21 treatment was discontinued because of insufficient effect, and in 3/21 because of side effects of skin rash, elevated liver transaminase levels and renal failure secondary to systemic hypotension. Discontinuation of bosentan following surgical progress was achieved in 2/21 (Fig. 5). No other major side effects were reported in the cohort. Saturations in 8 patients who had been weaned from bosentan were followed for 1 year. No statistically significant saturation changes were found in this group (*p* = 0.45). Saturations for patients weaned from sildenafil were not available.

## Discussion

In single ventricle physiology low pulmonary vascular resistance is one of the important factors which will influence functional ability, quality of life, and long-term survival [1]. It is established that a mean pulmonary arterial pressure > 15 mmHg or impaired ventricular function will increase the risk of single ventricle physiology failure after total cavopulmonary connection surgery [2, 3, 22, 23]. This has provided the rationale for increased use of pulmonary vasodilators in this patient group.

This retrospective analysis describes the effects of vasodilator therapy in 36 children with single ventricle physiology and pulmonary vascular disease. To our knowledge this represents one of the larger pediatric cohorts and the total follow-up time of 4.7 (0–12.8) years is longer than for previous publications.

Treatment with a single vasodilator, either sildenafil or bosentan, was associated with a significant improvement in saturation within 12 months after treatment initiation. The mean increase from 80 to 85% is moderate, but an indirect indicator of reduced pulmonary vascular resistance and increased pulmonary blood flow. We also observed a reduction in the mean pulmonary arterial pressure from 19 to 14 mmHg within a mean of 1.3 (range 0.3–2.6) years after treatment start. The clinical effect of the increase in saturation and reduction in pulmonary arterial pressure was harder to determine. Although the majority of patients reported improved well-being on treatment these reports were subjective.

There remains debate as to the mechanisms of pulmonary hypertensive vascular disease in patients with palliated univentricular hearts. On the one hand, there are mechanisms that induce high pulmonary vascular resistance. It has previously been shown that lack of pulsatile flow is associated with a decreased expression of important vasodilator substances, decreased vasorelaxation, and a vascular remodeling characterized by altered apoptosis of vascular smooth muscle cells [5, 24]. There is also an overexpression of the potent vasoconstrictor endothelin-1 in lung tissue of single ventricle patients [15]. Increased medial thickness and extension of the smooth muscle cells to the walls of distal intra-acinar pulmonary arteries in biopsies taken prior to total cavopulmonary connection have been shown to correlate with poor prognosis [25, 26]. On the contrary, Ridderboos et al.[6] found intimal fibrosis and reduction of muscular smooth cells in lung tissue obtained from autopsy in long-standing single ventricle physiology patients. A clear understanding of these mechanisms should deliver new treatment that may further reduce pulmonary vascular resistance.

A number of potential risk factors for high pulmonary vascular resistance were identified in our cohort. Late pulmonary arterial banding with excess circulation to the lungs was found in 11 patients and 10 patients had a restrictive atrial communication that required an intervention. Highly complex heart defects with isomerism and anomalous drainage of venous return were also common in the cohort. Prior to vasodilator therapy 79% (22/28 of known pressure values) of the patients in our study had elevated pulmonary arterial pressures.

Our results corroborate the findings of previous studies. Park et al. [18] retrospectively studied 34 patients and reported a significant reduction in mean pulmonary arterial pressure following vasodilator treatment. Mori et al. [17] followed 42 patients, whereof the majority were children, who were in different stages of single ventricle physiology and treated with sildenafil for 3 months. They too reported a significant reduction in mean pulmonary arterial pressure. Recently, Castaldi et al. studied effects of pulmonary vasodilators and could among other hemodynamic measures also show a reduction in pulmonary artery resistance [27]. Studies focused on total cavopulmonary connection in smaller cohorts have also shown positive early effects of sildenafil including increased saturation, reduced pulmonary arterial pressure, and reduced pleural effusion [20, 28]. Ovaert et al. [29] treated 10 single ventricle patients for 16 weeks with bosentan. They did not observe any significant improvement, but a trend towards an increase in saturations and reasoned that small numbers was a limitation for their study. Other smaller studies have reported favorable effects of bosentan on mean pulmonary arterial pressure, functional class, and 6-min-walk test [30, 31]. Handler et al. [32] showed significant decrease in pulmonary vascular resistance and increase in saturation with subcutaneous treprostinil for 17 children with single ventricle. A recently published large randomized controlled trial by Goldberg et al. demonstrated that udenafil compared to placebo had a modest positive effect on oxygen saturations and measures of submaximal exercise performance in adolescents after total cavopulmonary connection palliation, but udenafil was not shown to improve the primary outcome measure of peak oxygen consumption [33]. The patients in our cohort by comparison were far more symptomatic at baseline with a high proportion of risk factors for pulmonary vascular dysfunction and therefore may have a greater potential for benefit. In their initial report Goldberg et al. have not reported whether a subgroup of more symptomatic patients sustained greater benefit.

In our study, sildenafil was the first choice in most cases when a child was started on pulmonary vasodilator therapy. Bosentan was in most cases chosen as an add-on when a second vasodilator was considered necessary. This practice mirrors those reported from other registries [34, 35].

We did not observe any consistent additional changes in saturation and mean pulmonary arterial pressure when a second vasodilator was added. This lack of response with add-on therapy was also seen in the COMPASS-2 study where added bosentan was not superior to sildenafil monotherapy in delaying the time for the first morbidity/mortality event in patients with pulmonary arterial hypertension and biventricular physiology [36]. On the other hand, initial combination therapy has shown reduced risk of clinical failure events in the recent published study by White et al.[37]. In our study, a few patients with single ventricle physiology were given initial combination therapy, but the numbers were too small for comparison with monotherapy.

Few adverse effects were observed during treatment or after discontinuation. With a total follow-up time of 4.7 years (0–12.8), this represent one of the longer observations. In this cohort both sildenafil and bosentan were well tolerated.

There are limitations to this observational study. The United Kingdom Service for Pulmonary Hypertension in Children is a national referral center. The findings in this study are therefore vulnerable to referral bias. Many of the patients were already treated with sildenafil when first seen by the service. They may also represent a selection of more complex single ventricle physiology compared to those seen at a local center. Despite a large center the number of children with single ventricle physiology treated with these drugs were small. Some patients had to be excluded from the analysis because of confounding factors (see methods). This study has no control group, as all patients followed by the United Kingdom Service for Pulmonary Hypertension in Children had an indication for vasodilator therapy and all were treated. The heterogeneity of the cardiac diagnoses in this group made it difficult to predict who would benefit from vasodilator treatment.

In conclusion, vasodilator therapy can achieve and sustain improvements in both saturation and mean pulmonary arterial pressure in a group of severely affected children with single ventricle physiology. The total mean follow-up time was 4.7 years. There were no major adverse reactions with long time vasodilator therapy and vasodilators could be discontinued safely when indicated. 7/12 patients progressed in surgical stage during treatment. This study adds useful information on long-term effects of pulmonary vasodilators in a group of complex single ventricle patients with suspected pulmonary vascular disease. The cohort is also one of the largest so far, with longer total follow-up time compared to previous publications. The mechanisms for pulmonary vascular disease in these children are not fully understood and further studies, ideally experimental studies and prospective multicenter randomized controlled trials, are needed.
